# Effect of Abutment Height on Retention of Single Cement-retained, Wide- and Narrow-platform Implant-supported Restorations

**DOI:** 10.5681/joddd.2012.021

**Published:** 2012-09-01

**Authors:** Fariba Saleh Saber, Nader Abolfazli, Sara Nuroloyuni, Sohleh Khodabakhsh, Mehran Bahrami, Reza Nahidi, Somaieh Zeighami

**Affiliations:** ^1^Associate Professor, Department of Prosthodontics, Faculty of Dentistry, Tabriz University of Medical Sciences, Tabriz, Iran; ^2^Associate Professor, Department of Periodontics, Faculty of Dentistry, Tabriz University of Medical Sciences, Tabriz, Iran; ^3^Post-graduate Student, Department of Pediatric Dentistry, Faculty of Dentistry, Tabriz University of Medical Sciences, Tabriz, Iran; ^4^Dentist, Private Practice, Urmieh, Iran; ^5^Assistant Professor, Department of Prosthodontics, Faculty of Dentistry, Tehran University of Medical Sciences, Tehran, Iran

**Keywords:** Dental abutments, dental implants, implant-supported prosthesis, retention

## Abstract

**Background and aims:**

In contrast to prepared natural dentin abutments,little is known concerning factors influencing the retention of fixed prostheses cemented to implant abutments. The aim of this study was to investigate the effect of im-plant abutment height on the retention of single castings cemented to wide and narrow platform implant abutments.

**Materials and methods:**

Thirty-six parallel-sided abutments (Biohorizon Straight Abutment) of narrow platform (NP) and wide platform (WP) sizes with their analogs were used. In each group of platform size, abutments were prepared with axial wall heights of 5, 4, 3, 2 mm (n=9). On the whole 72 castings were constructed, which incorporated an attachment to allow removal. Castings were cemented to abutments with TempBond®. A uniaxial tensile force was applied to the crown using an Instron machine until cement failure occurred. Analysis of variance of the models were fit to determine the effect of height of abutment of the restorations on the mean tensile strength (α=0.05).

**Results:**

The mean peak removal force for corresponding abutments was significantly different (P < 0.05): (1) with plat-form sizes: WP > NP; (2) with alteration of axial wall height for NP: 5 mm > 4 mm > 3 mm = 2 mm and for WP: 5 mm > 4 mm = 3 mm = 2 mm.

**Conclusion:**

The retention of NP cement-retained restorations is influenced by the wall height but not in same manner as WP. Restorations of narrow-platform size with longer abutment exhibited higher tensile resistance to dislodgement.

## Introduction

Implant designs, surface modifications and successful osseointegration of implant materials and soft tissue management techniques have allowed the single tooth screw-retained implant procedure to become a viable treatment option.^1 - 5^

The retrievability of implant abutment restorations is essential for the maintenance of implants and repair of the prosthesis.^6^ Screw-retained, implant-supported prostheses were developed in response to the need for retrievability of restorations. Initial clinical investigations of single-tooth screw-retained restorations showed that loosening of the retaining screw that fixes the prosthesis to the implant abutment is a common problem. However, this complication not only does not affect the survivability of the implant ^7- 9^ but has allowed the development of cemented implant-supported restorations. In fact, the use of cement-retained, implant-supported restorations has increased, due in part to the ability to optimize occlusal interdigitation, enhance esthetics, provide a passive fit, decrease the cost, and improve loading characteristics.^9^

The success of cement-retained designs depends largely on adequate retention and resistance.^10^ There are many factors that can influence the amount of retention that can be achieved when luting a restoration to either an abutment or a natural tooth.^11^ Factors affecting implant supported restorations are similar to those affecting the luting of crowns to natural teeth, and include taper, height, width of the abutments, and the type of luting agent. For patients with limited interocclusal space, shorter abutments might be desirable and lack of retention has been shown to be a common cause of fixed prosthesis failure.^12^ Accepted techniques for improving the retention of a restoration include increasing the size, surface area, occlusogingival preparation height, parallelism of opposing walls, controlling taper,^13 - 16^ and also making retentive guiding grooves.^17 - 21^ Most studies have used die materials that do not have the characteristics of implant abutment structure. Evaluation of restoration retention using standardized abutment would provide valuable clinical information.

These findings indicate that many questions remain in determining the factors influencing retention. The aim of this study was to determine the effect of the height of implant abutment on retention of single cement-retained, wide- and narrow-platform implant-supported restorations. The null hypothesis was that there would be no difference in retention between different heights of implant abutments when using narrow and wide platforms.

## Materials and Methods

In order to evaluate the effect of abutment height on retention of cement-retained single restorations, parallel sided abutments (Biohorizon Straight Abutment, Birmingham) were selected in this study as they are preformed standardized abutments with zero degree angle walls. Thirty-six narrow- (3.5 mm) and wide-platform (5 mm) abutments were attached to their implant analogs and vertically mounted in acrylic resin (RP self-cured clear acrylic resin, Dentsply DeTrey GmbH, Konstanz, Germany) to permit a tensile force to be applied in the long axis of the abutment. The perpendicular placement of the implant analogs in the resin blocks was verified with a dental surveyor (Ney Dental Intl, Bloomfield, CT, USA). The abutments were connected to the implant analogs and torqued to 30 N/cm. A pilot study was conducted to determine the abutment heights investigated. From this data, a 5-mm occluso-gingival height was established as the control because these samples had the same retention characteristics as samples with greater heights. Three experimental groups were selected with heights of 4.0 mm, 3.0 mm, and 2.0 mm of each platform size. Nine samples in each group were tested for retention. The screw access hole of the abutment was covered with a cotton pellet, and the access hole was closed with Cavit (3M ESPE, St. Paul, MN) flush with the occlusal surface of each abutment. Two layers of die spacer (Belle de St Claire, Kerr Laboratories, Orange, CA) were painted directly to the abutments within 0.5 mm of the margin. A wax coping with 1.5 mm average thickness for each sample was fabricated with a direct wax technique. A U-sprue was waxed to the upper surface of coping to permit the connections necessary to engage a special device of the Instron machine. The 72 wax patterns were invested and cast in base metal alloy (Rexillium III, Pentron Laboratory Technologies, Wallingford, CT) according to manufacturer’s instructions. To assess standardization of copings, the copings were interchanged with different abutment samples and evaluated for adaptation and accuracy of fit. Visual inspection was carried out at ×10 magnification for nodules and clinical examination was carried out with an explorer for evaluation of marginal integrity. Passivity of fitness of copings were checked with silicon fit-checker (GC America, Inc., USA) on their respective abutment. Copings were accepted when they seated completely with no gap along the entire margin. Nine castings for each height of abutment were cemented to the abutments by weighed amounts of TempBond (Kerr Italia S.P.A, Scafati, Italy) provisional cement which was mixed for 30 seconds according to manufacturer’s instructions. The castings were filled with mixed cement using a crown-filling technique, seated with finger pressure, and placed under a 10-kg load for 5 minutes.^22^ The cemented copings were placed under 100% humidity at 37°C for 24 hour before testing. A universal load-testing machine (Instron, Norwood, USA) was used to measure the peak force required to remove the castings from the abutments with a crosshead speed of 5 mm/min until cement failure occurred.^11 , 15 , 16^

Differences in mean tensile strength were analyzed by two-way ANOVA (α = 0.5). A conservative post hoc test correction was applied to evaluate differences between the means of subgroups. Statistical significance was defined at P < 0.05.

## Results

The mean tensile bond strength values (±SE) or the force required to remove the coping from the abutment after cementation for the four different heights of each platform size are shown in Table 1. The retention forces for narrow-platform abutments (NP) ranged from 10.11±2.7 N (height: 2 mm) to 34.68 ± 0.77 N (height: 5 mm), and for wide-platform (WP) abutments from 9.92 ± 4.11 N (height: 2 mm) to 48.15±0.02 N (height: 5 mm). Based on ANOVA (F = 113.36, P = 0.001) and according to HSD Tukey test, the amount of force required to remove the coping from the abutment after cementation increased with an increase in the height of NP and WP abutments. In NP abutments there were statistically significant differences between all the heights with one exception between heights 2.0 and 3.0 mm; however, in the WP abutments there were statistically significant differences in mean retention only between heights of 4.0 and 5.0 mm (Table 2).

**Table 1:** Mean tensile bond strength values for the four different heights of each platform size

		Narrow platform		Wide platform	
Comparative heights		Mean difference ± SE	P value	Mean difference ± SE	P value
2	3	-3.18 ± 1.48	0.159	-11.16 ± 1.96	0.060
2	4	-10.92 ± 1.48	0.000*	-17.52 ± 1.91	0.000*
2	5	-24.57 ± 1.44	0.00*	-38.23 ± 1.91	0.000*
3	2	3.18 ± 1.48	0.159	11.16 ± 1.96	0.060
3	4	-7.73 ± 1.52	0.000*	-6.35 ± 1.86	0.101
3	5	-21.39 ± 1.48	0.000*	-27.06 ± 1.86	0.000*
4	2	10.92 ± 1.48	0.000*	17.52 ± 1.91	0.000*
4	3	-10.92 ± 1.48	0.000*	6.35 ± 1.86	0.101
4	5	-13.65 ± 1.48	0.000*	-20.71 ± 1.81	0.000*
5	2	24.57 ± 1.44	0.000*	38.23 ± 1.91	0.000*
5	3	21.39 ± 1.48	0.000*	27.06 ± 1.86	0.000*
5	4	-13.65 ± 1.48	0.000*	20.71 ± 1.81	0.000*

P values from ANOVA and post hoc test. *Statistically significant

**Table 2:** Mean and comparative retentive forces between wide and narrow platform size

Comparative heights	Mean difference ± SE	P value
2 mm		
Wide platform	9.92 ± 1.37	0.90
Narrow platform	10.11 ± 0.86	
3 mm		
Wide platform	21.08 ± 1.15	0.00*
Narrow platform	13.29 ± 1.35	
4 mm		
Wide platform	27.44 ± 1.94	0.21*
Narrow platform	21.03 ± 1.46	
5 mm		
Wide platform	48.15 ± 0.00	0.00*
Narrow platform	34.68 ± 0.02	

P values from ANOVA and post hoc test. *Statistically significant

The comparative retentive forces to remove the cemented copings from the abutments were always as follows: WP>NP with one exception in 2-mm height.

## Discussion

The null hypothesis of this study that there would be no difference in retention of single cement-retained restorations between different heights of implant abutments with the use of narrow- and wide-platforms was partially rejected. The results of this study showed that varying height had significant influences on retention of NP single cement-retained restorations but not in the same manner as that with WP ones. In other words, increasing NP abutments’ height and the height-to-width ratio had a positive effect on the retention of WP abutment cemented restorations and preparation heights of 3 mm for NP and 4 mm for WP are the minimum abutment height necessary to provide adequate retention; however, the limitations of this study should be noted from the outset since it only investigated retention and not resistance.

Absence of a standardized test for determining the retention of restorations to abutments and differences in specimen preparation and study methods prevent exact comparison of results with those of other studies. However, this study as well as that by Kent et al^11^ indicate the effect of abutment height on restoration-to-abutment retention. Maxwell et al^23^ later found similar relationship for height of abutment and concluded that at 6-degree taper, 3 mm was the minimal height for adequate retention in natural teeth. The results of a study by Kaufman et al^24^ demonstrated a linear relationship between retention and preparation height, but the minimum preparation studied was 4 mm.

Data from this study and a study by Bernal et al^13^ provide information concerning the effect that abutment height has on retention of a restoration. It seems the effect of abutment height on the retention of NP single restoration is similar to natural tooth samples.

In the present study, the comparative tensile forces required to remove the cemented coping from the abutment were significantly higher for wide-platform than narrow-platform abutments with one exception at height of 2.0 mm. This does not imply that narrower abutments are not appropriate for cemented restorations; rather, at some levels the abutment height may become an issue if shorter abutments are used. This confirms current understanding that retention decreases with a decrease in diameter. ^24 , 25 , 26 - 28^ Clinically, this means that if the retentive form of the abutment is compromised, through for example limited interocclusal space and occlusion,^13^further caution should being exercised to ensure that other retentive features of the abutment are maximized. Conversely, one of the major concerns with cemented restorations is the challenge of retrieval when an abutment screw loosens. A casting for an implant abutment may be difficult, if not impossible, to retrieve without sectioning it.^13^ Clinically, if there is a risk of screw loosening and the retentive form of the abutment is high, the results of this study suggest that height reduction of the NP abutments can have a significantly detrimental effect on retention form of an abutment.

It is interesting to note that the retentive strength values of WP implant abutment restorations in the present study were approximately one-third those of the prepared natural tooth abutments used in a study by Breeding et al.^10^ It seems that axial loads at failure achieved with the WP implant abutment restorations exceeded the loads generated by cast restorations cemented to natural abutments. Results of the present study and those by Kent et al^11^ and the Covey et al^14^ provide information about the effects of abutment size on crown-to-abutment retention: increasing the abutment’s vertical height or the height-to-width ratio has a positive effect on tensile testing values of cemented restorations.

According to the result of the present study, there was a significant increase in retention of WP implant abutments with an increase in abutment height from 2 to 3 and 3 to 4 mm were. Wide abutments with the greatest total surface area did not exhibit improved crown retention when compared with narrow abutments. Darveniza et al^2^ used natural abutments and reported a similar relationship between width and retention. However, this result is in contrast to the findings by Kaufman et al,^24^who reported that increasing the diameter of tapered dies along with increasing vertical height results in significant increases in the crown-to-die retention, which might be attributed to differences in geometry between natural and implant abutments and in the amount of taper. This study showed that vertical height of NP implant abutment and the ratio of vertical height-to-width of WP implant abutment influence the amount of retention provided in a cement-retained restoration. Total surface area and width of the abutment do not provide good predictors of uniaxial retention values.

One of the limitations with this study was that it evaluated the effect of abutment height on the retention of restorations, which is one of the factors influencing retention. It is recommended that further research be undertaken on other factors such as convergence angle, cement type and abutment surface texture. In fact, it only investigated retention rather than resistance. Clinically, removal of castings might not employ forces along a single path of withdrawal.

## Conclusion

Within the limits of this study, the following conclusions were drawn:

1. Varying implant abutment height had a significant effect on retention of NP single cement-retained restorations but not in the same manner as that with WP single cement-retained restorations.
2. The minimum abutment heights necessary to provide adequate retention for NP and WP single cement-retained restorations were 3 mm and 4 mm, respectively.
3. Abutment height and height-to-width ratio of WP single cement-retained restorations were positively related to retention strength, whereas an abutment’s total surface area and width were not.
